# Does Being Ill Improve Acceptance of Medical Technology?—A Patient Survey with the Technology Usage Inventory

**DOI:** 10.3390/ijerph18179367

**Published:** 2021-09-05

**Authors:** Sabur Safi, Gerhard Danzer, Solaiman Raha, Eyyad Nassar, Frank T. Hufert, Kurt J. G. Schmailzl

**Affiliations:** 1 Fakultät Humanwissenschaften, Medical School Hamburg, Am Kaiserkai 1, 20457 Hamburg, Germany; 2 Fachbereich für Innere Medizin und Psychosomatische Medizin, Allgemeine Psychologie, Medical School Brandenburg, Fehrbelliner Str. 38, 16816 Neuruppin, Germany; gerhard.danzer@mhb-fontane.de; 3 Fachbereich für Digitale Medizin und Künstliche Intelligenz, Center for Connected Health Care UG, Gartenstrasse 20, 16818 Wustrau, Germany; solai@connectedhealth.com.de (S.R.); eyyad@hotmail.com (E.N.); ccc@connectedhealth.com.de (K.J.G.S.); 4 Fachbereich für Mikrobiologie und Virologie, Medical School Brandenburg, Fehrbelliner Str. 38, 16816 Neuruppin, Germany; frank.hufert@mhb-fontane.de

**Keywords:** COVID-19, technology acceptance, user survey, wearable health monitor, ECG patch

## Abstract

Acceptance of new medical technology may be influenced by social conditions and an individual’s background and particular situation. We studied this acceptance by hypothesizing that current and former COVID-19 patients would be more likely to accept an electrocardiogram (ECG) “patch” (attached to the chest) that allows continuous monitoring of the heart than individuals who did not have the disease and thus the respective experience. Currently infected COVID-19 patients, individuals who had recovered from COVID-19, and a control group were recruited online through Facebook (and Instagram) and through general practitioners (GPs). Demographic information and questions tailored to the problem were collected via an online questionnaire. An online survey was chosen in part because of the pandemic conditions, and Facebook was chosen because of the widespread discussions of health topics on that platform. The results confirmed the central hypothesis that people who had experienced a disease are more willing to accept new medical technologies and showed that curiosity about new technologies and willingness to use them were significantly higher in the two groups currently or previously affected by COVID-19, whereas fears of being “monitored” (in the sense of surveillance) were significantly higher among people who had not experienced the disease and threat. Experiencing a serious disease (“patient experience”) promotes acceptance of new medical technologies.

## 1. Introduction

People’s attitudes toward new medical technologies are driven in part by their cultural background and their confidence in the existing health care system and its effectiveness and equity. However, there is a two-fold problem regarding medical technology—its availability and, perhaps more critically, people’s acceptance of technology in general and their willingness to accept monitoring or even accompanying inconveniences caused by new, unfamiliar technologies. The Unified Theory of Acceptance and Use of Technology (UTAUT) is a theory used to predict consumer acceptance of technology. It assumes four independent factors influence acceptance [1,2]. These four factors are performance expectancy, effort expectancy, social influence and facilitating conditions.

This theory, originally formulated in computer science, can also be applied to predicting user acceptance of medical technology. As in computer science, the acceptance of medical technology by its users, consumers, and patients depends on many different underlying conditions. These include device features (performance expectation of the technology), ease of use, costs, whether the medical device constrains its user or how this constraint relates to the expected benefits of the device (effort expectation), and appearance and other socially determined features and circumstances [3]. These conditions are significantly shaped by the patient’s overall background, including their medical history, and their current health status.

For example, GPS trackers are commercially available for older people with mild to moderate dementia to allow them to go out on their own and, should they become lost, to be found easily. GPS trackers can be used for many purposes outside of medicine, but can be used in medicine for people with cognitive impairment and dementia-related syndromes. Caregivers or family members involved in the care can use a smartphone app to locate the patient is in real time. This allows the person being monitored to actually be more independent. At the same time, however, it is possible that the monitoring function may be perceived as an invasion of privacy, and this, along with a threshold fear of the new technology, may result in the tool not being adopted. Threshold anxiety is a term used to explain the psychological barriers people experience, or the fear people feel when faced with something unknown, in this case a new technology. Acceptance depends on how the patient views the advantage of the device, in this case the ability to go out alone, versus the disadvantage of the impairment caused by the device [4,5].

The outcome of this cost–benefit balance depends on the particular circumstances. In the case of the GPS tracker, the average healthy user would most likely feel they were subject to excessive surveillance. In contrast, for a patient with cognitive impairment, the tracker may provide a level of security that is perceived as reassuring and/or makes unaccompanied walks possible, i.e., expands rather than restricts the user’s freedom [3]. Therefore, technologies can be useful in the medical field for a variety of purposes.

Numerous other examples show that patients are more likely to accept a medical device that would be completely unacceptable to healthy individuals because they expect to receive benefits in their situation that would otherwise not be available [6,7,8]. These include technologies for detecting falls or epileptic seizures, continuous blood glucose monitors, and other more or less invasive devices for diabetics [9,10,11]. Acceptance of reusable respiratory filters increased in the wake of the current COVID-19 pandemic [12]. As more people began to use different types of face masks, the threat of shortages became greater and some countries even stopped the export of face masks so they were able to meet the demand within their countries [13]. Consequently, it can be deduced that there is a correlation between the demand and the shortages, and between the more widespread use of face masks and the higher demand for them.

It is therefore evident that the acceptance of a medical device depends on the individual and changing circumstances of the user. However, there is little literature that has directly compared the acceptability of a medical device in patients and healthy individuals or has investigated whether healing from an acute illness affects acceptability. For this reason, the pilot study presented here was conducted. This study can be a step forward in filling the research gap in the area of the relationship between the medical condition and patients’ acceptance of new medical technology. In the present paper, patients with active COVID-19 disease (Group 1—COVID), people who had survived COVID-19 disease (Group 2—recovered), and Group 3—healthy people (regardless of the possible presence of other comorbidities) were asked about their acceptance of medical technology using the example of an electrocardiogram (ECG) patch. Acceptance of medical technology is important for several reasons. First, medical technology can improve the likelihood of recovery for people who are overcoming illnesses that need further medical support. Second, medical technology can help in the diagnosis and early detection of illnesses. Finally, medical technology can help in monitoring medical conditions. Consequently, acceptance of medical technology can significantly improve the overall medical health of people at the different stages of a medical condition—either for prevention, recovery, or monitoring. The present study aimed to compare the attitude towards medical technology in general, and towards the electrocardiogram (ECG) patch in particular, of healthy people, people suffering from COVID-19, and people who have survived COVID-19. The study maintained the thesis that being a patient, that is, suffering from a medical condition or illness, increases the level of acceptance of medical technology. The study is innovative in the manner that it collects data from healthy people, which was not undertaken in most of the previous research. Collecting and analyzing data from healthy people is important in evaluating the general attitude towards medical technology and not only the attitude of those suffering from an illness. By being aware of healthy people’s attitudes towards new medical technology, we can bring about an overall positive change by educating healthy people about the importance of medical technology. It is clearly preferable that this change in attitude occurs prior to individuals becoming ill, given the time it takes to bring about a change in their acceptance may prove fatal.

## 2. Material and Methods

### 2.1. Recruitment

The survey was conducted online from April to November 2020 using Unipark academic survey software, that runs on Questback EFS (Winter 2019 v.32.2). Unipark was the preferred method for data collection due to its user-friendly interface, which ensured positive participant attitudes toward the survey, and its flexibility in creating question types and structure. Overall, the method ensured that data would be collected objectively and securely, because Unipark offers a high level of data security. In addition, Unipark provides the ability to extract data in a variety of formats. For the present study, the data were stored in Excel. Overall, Unipark provided participant satisfaction and ease of subsequent analysis of the data collected. The survey was placed on Facebook in various COVID-19 groups, and participants were asked to respond anonymously. Data collection took place in Germany and neighboring countries, because the COVID-19-related groups in which the survey was distributed included individuals from these countries. Data were collected anonymously and voluntarily, and the analysis did not include an assessment of the particular situation or condition of individual participants, but simply their opinions on medical technology issues. Therefore, the survey posed an extremely low risk to the participants and the data they provided, and participant consent was not required. The data collected were not used to link them to individuals, nor was it of interest to determine who the individuals were. In addition, two bloggers on Instagram helped distribute the survey and two general practitioners (GPs) distributed the survey to their respective patients. This aimed to reach many and varied people, and recruit them to complete the survey. The survey distributed by the bloggers and the GPs was not different from the other survey posted in the Facebook groups. In each case, the survey was distributed via a link and subject participation was voluntary and anonymous. Study participants were divided into three study groups: (a) COVID-19 patients with active symptoms, (b) individuals who had recovered from COVID-19, and (c) healthy individuals without current and previous COVID-19 disease. The inclusion criterion was a clinically confirmed diagnosis, i.e., COVID-19 confirmed by PCR assay. Exclusion criteria included misunderstanding the purpose of the survey and lack of access to technology, which effectively meant that these individuals could not participate in the survey. Patients with severe cases of COVID-19 who were being treated in an intensive care unit and were unable to actively participate were also excluded from the survey.

### 2.2. Data Collection

All study participants were given a questionnaire, included in the Appendix A, to collect basic demographic data and data on their attitudes toward technology in general, and finally to collect data on their attitudes toward technology in light of the current COVID-19 pandemic. The questionnaire started by collecting general demographic and social data such as gender, age, place of residence, marital status, and current health status in relation to COVID-19 (whether the person has had it before, currently has it, is hospitalized, or has never been exposed to the disease). The following questions about participants’ general attitudes toward technologies and technology aimed to measure the level of understanding of new technologies, whether participants see technology as a facilitator and a means to make daily life easier, how affordable they think new technology is and should be, and whether they associate technologies with dangers and risks. The questions deal with how they learn about new technologies. Each question was answered on a seven-point Likert scale, with half points also possible as an answer. The following ten questions on COVID-19 contained the same scale. The questions aimed to measure correlations between whether a person is or has been affected by COVID-19 and his or her attitude toward technology and technical aids, and any change in attitude toward medical technology resulting from the individual being affected. The questionnaire also elicited attitudes toward the eight general factors of curiosity, fear of technology, interest, ease of use, usefulness, skepticism, accessibility, and “intention to use”. The questionnaire was based on the Technology Usage Inventory (TUI), although one dimension of the instrument (intention to use) was omitted because it was not relevant to this case study.

In addition, ten other questions developed by the authors were included that aimed to measure attitudes specifically of individuals who had been exposed to COVID-19.

All participants completed the survey online. However, the number of individuals >65 years of age who completed the survey was lower. Nonetheless, the data collected from individuals >65 years of age were included in the data set and were not treated as a subgroup despite the lower number of participants.

### 2.3. Statistical Analysis

The dataset was analyzed using SPSS (Statistical Product and Service Solutions), first looking for relationships between participants and the expressions of each variable. Following this, the variables were analyzed among themselves. This included the internal consistency (Cronbach’s alpha) and the discriminatory power of the items (ITC = item-total correlation); a value of >0.7 was considered acceptable. For the discriminatory power, the correlation between the measured value of an item and the result of the measured value of the variable without the respective item under consideration was determined. Items with a correlation of r < 0.3 were excluded. For the analysis of the present data, the TUI was a particularly appropriate instrument because it provides a different scale for assessing both general attitudes toward technology and attitudes toward a specific device.

For the descriptive analysis, the median and the 25% and 75% quartiles, the mean, the standard deviation, and the minimum and maximum values were calculated. Histograms of response frequencies were created for graphical representation. Differences between the means of the three study groups were tested for significance using ANOVA, and a *p*-value of *p* < 0.05 was considered significant.

## 3. Results

### 3.1. Basic Demographic Parameters

This study was conducted to test the hypothesis that there is a correlation between experience of a condition and attitudes toward medical technology.

The study compared attitudes toward medical technology in general and, specifically, acceptance of an ECG patch in a group of current COVID-19 patients (COVID), a second group who had survived COVID-19 (COVID-recovered), and healthy individuals (healthy).

A total of 607 participants took part in the study, including 155 men (25.5%). The COVID group included 130 participants, of whom 70 (53.7%) were male. The COVID-recovered group consisted of 127 participants, of whom 59 (46.5%) were male. Group 3, in which participants had no personal current or previous experience with COVID-19, consisted of 350 participants, of whom 26 (7.4%) were male. Four (0.7%) study participants reported “diverse” as their gender (one each in the COVID- and COVID-recovered groups and two in the healthy group). Thus, the healthy group had a statistically significant excess of females compared to the other two study groups (*p* < 0.001). The two COVID groups also differed from group 3 in terms of age distribution. Group 3 had a significantly higher proportion of study participants in the 18–24 and 25–34 age groups, whereas Groups 1 and 2 had a higher proportion of participants in the 35–44 and 45–54 age groups, respectively (*p* < 0.001).

Other baseline demographic parameters of the study participants are listed in Table 1. Significant differences between groups existed in terms of marital status, smoking status, education level, and place of residence, in addition to gender and age. Groups 1 and 2 were relatively similar to each other and different from Group 3, but the groups did not differ with respect to the proportion of study participants with minor children (*p* = 0.380).

### 3.2. Technology Acceptance

The study showed that a significant difference exists among the three groups in terms of various acceptance parameters for the ECG patch. Thus, scores for both curiosity about the technology and interest in using it were highest in the COVID group and lowest in the healthy group, with group differences reaching significance only for curiosity (A, *p* < 0.001 and B, *p* = 0.739). Conversely, an opposite tendency was found for fear of technology and skepticism toward technology. Here, the mean values were highest in the healthy group and low in COVID-19 patients and those who had recovered from COVID-19. Again, group differences reached significance for only one of the two factors, skepticism (C, *p* = 0.148 and D, *p* = 0.001). The COVID group also had the highest expectations concerning ease of use (*p* = 0.003), accessibility (*p* = 0.001), and usefulness (*p* = 0.005) of the ECG patch. Here, too, the recovered study participants had values that lay very close to those of the COVID group, whereas the values of the healthy people were much lower.

### 3.3. COVID-Specific Questions

**Figure 1:**
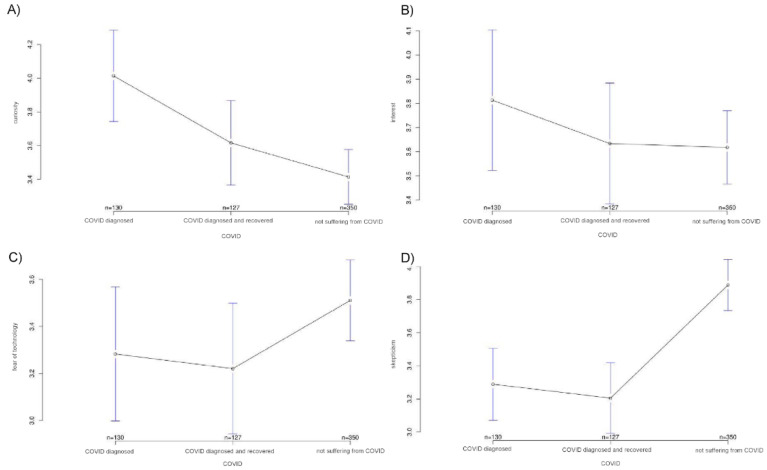
Average attitudes toward different aspects of technology acceptance in the three study groups. The *y*-axis shows the mean result of participants choice on the Likert scale: (**A**) Curiosity about the technology; (**B**) Interest in using it; (**C**) Fear of technology; (**D**) Skepticism about the technology.

**Figure 2:**
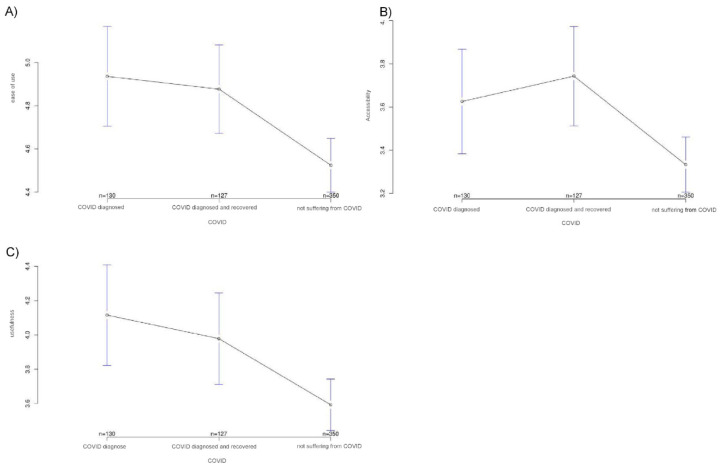
Expectation scores related to ease of use (**A**), accessibility (**B**), and usefulness (**C**) of the ECG patch in the three study groups. The *y*-axis shows the mean result of participants choice on the Likert scale.

The third part of the study examined how attitudes toward modern medical technology have changed as a result of the COVID-19 pandemic. A questionnaire with 10 questions was set up for this purpose. Again, there were some significant differences between the groups. Interestingly, the recovered group took an intermediate position between the diseased and healthy groups on five of these questions, showing surprisingly high or low scores in three cases (Figure 1 and Figure 2).

**Figure 3:**
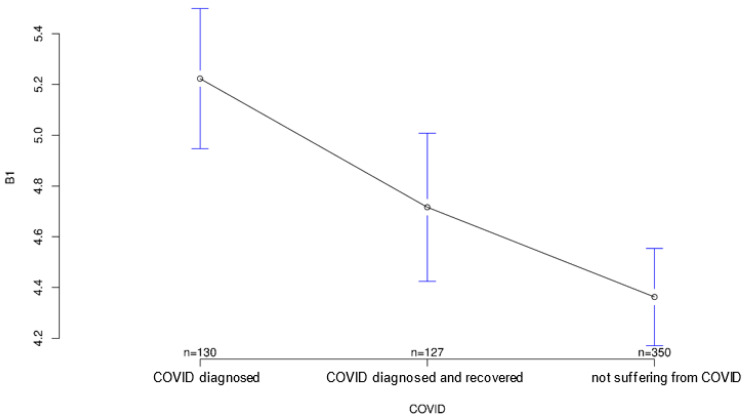
Average agreement with statement 1 of the COVID-specific questionnaire: “The Covid-19 pandemic is a reason for outpatient use of medical devices to monitor health conditions.” The *y*-axis shows the mean result of participants choice on the Likert scale.

**Figure 4:**
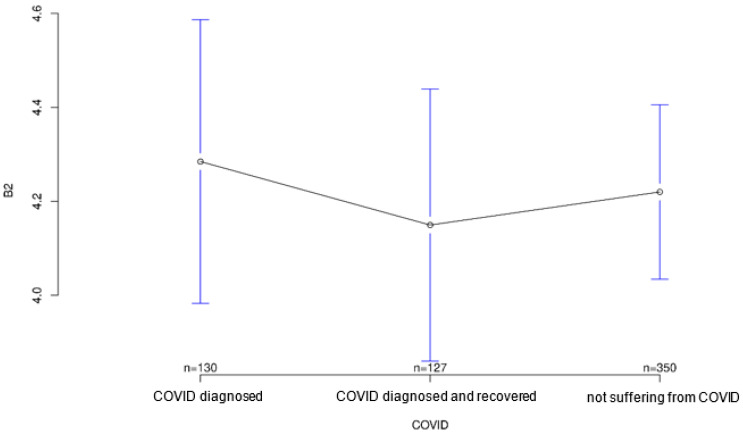
Average agreement with statement 2 of the COVID-specific questionnaire: “Multiple sensors on the body could make a person appear older or more ill than they are.” The *y*-axis shows the mean result of participants choice on the Likert scale.

**Figure 5:**
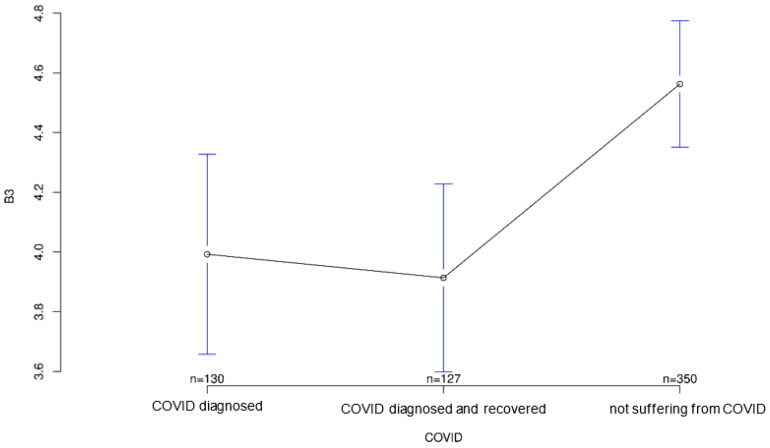
Average agreement with statement 3 of the COVID-specific questionnaire: “Body-worn sensor technology would always remind me of illness and thus would certainly be a psychological burden.” The *y*-axis shows the mean result of participants choice on the Likert scale.

**Figure 6:**
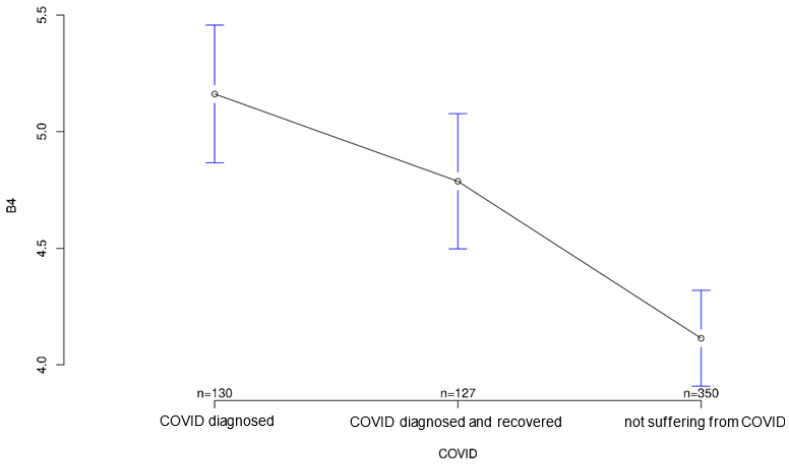
Average agreement with statement 4 of the COVID-specific questionnaire: “Body-worn sensor technology for monitoring could help reassure family and friends as my health would always be medically monitored.” The *y*-axis shows the mean result of participants choice on the Likert scale.

The group acutely affected by COVID-19 agreed most strongly with the statement that the COVID-19 pandemic could be a major reason for ambulatory use of medical devices to monitor their health status, followed by the recovered group (*p* = 0.001; Figure 3). Interestingly, however, both COVID-affected and healthy individuals on average agreed more strongly than the recovered group with the statement that multiple sensors on the body could make a person appear “older” or “sicker” than they are (*p* = 0.823; Figure 4), although group differences on this statement did not reach statistical significance. In contrast, both COVID patients and convalescents appeared to be more open-minded and tolerant of body-worn sensory technology and showed generally positive attitudes toward modern medical technology, but at the same time agreed significantly less often and less than Group 3 (healthy individuals) with the statement that such wearables would always remind them of “sickness” and thus would certainly establish mental distress (*p* = 0.001; Figure 5). The somewhat opposite statement that wearables and sensor technology could help reassure family and friends was answered accordingly by the three groups, with the COVID group having the highest mean, followed by the recovered group (*p* < 0.001; Figure 6).

**Figure 7:**
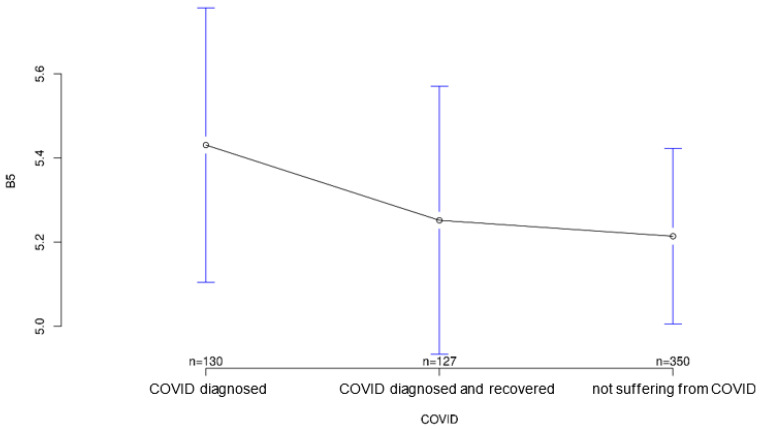
Average agreement with statement 5 of the COVID-specific questionnaire: “It’s important to me that such sensor technology looks aesthetically pleasing or is completely unobtrusive.” The *y*-axis shows the mean result of participants choice on the Likert scale.

**Figure 8:**
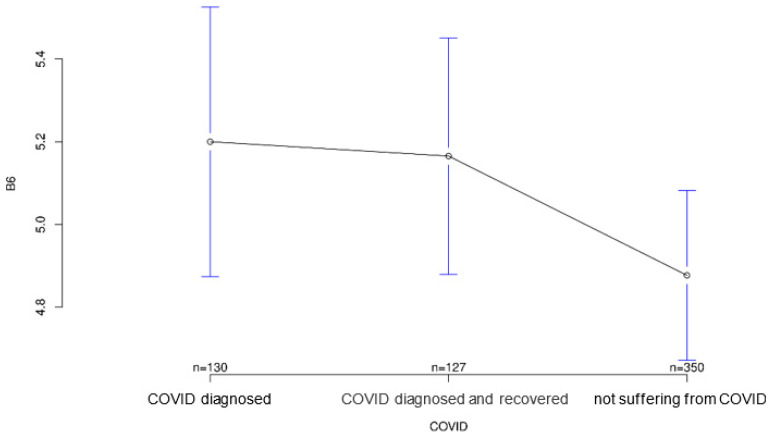
Average agreement with statement 6 of the COVID-specific questionnaire: “Thanks to new digital technologies, large amounts of data can be stored and retrieved at any time. My medical records should be available to my treating physicians.” The *y*-axis shows the mean result of participants choice on the Likert scale.

**Figure 9:**
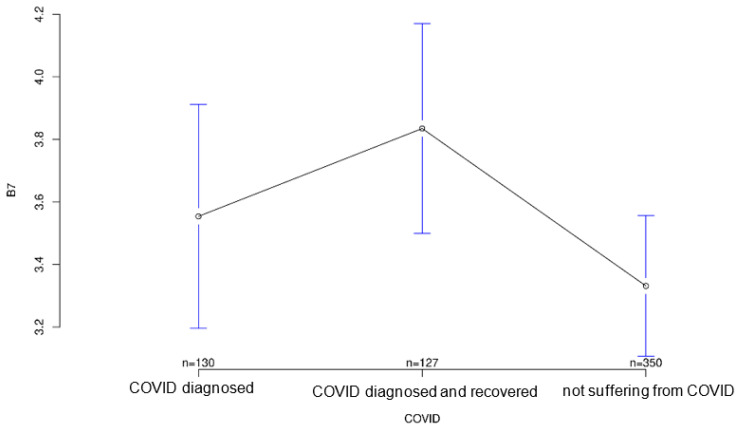
Average agreement with statement 7 of the COVID-specific questionnaire: “In the fight against pandemics, movement data can help—for example, to trace chains of infection. Medical data collected through body-worn sensor technology should be available to government institutions.” The *y*-axis shows the mean result of participants choice on the Likert scale.

**Figure 10:**
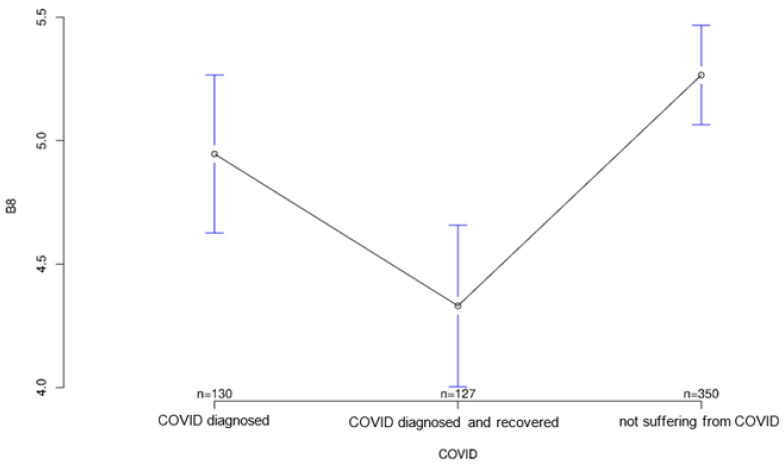
Average agreement with statement 8 of the COVID-specific questionnaire: “I have security concerns (e.g., hacking attacks/manipulation) about such sensor technology to monitor my health.” The *y*-axis shows the mean result of participants choice on the Likert scale.

**Figure 11:**
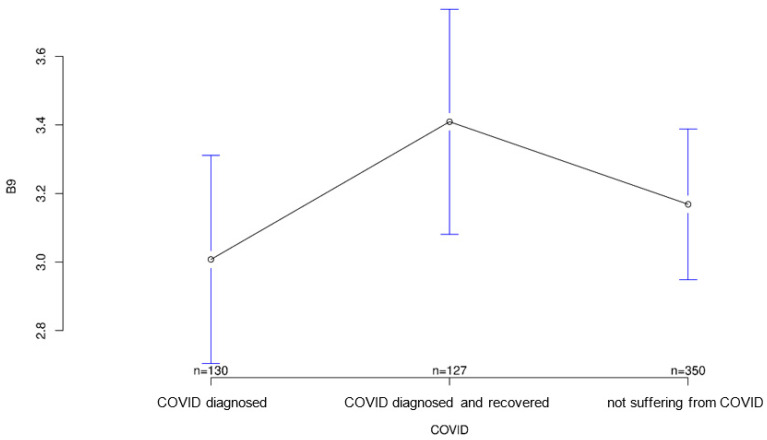
Average agreement with statement 9 of the COVID-specific questionnaire: “If health insurance companies do not cover the entire cost of such sensor technology, the cost should be offset by private co-payments.” The *y*-axis shows the mean result of participants choice on the Likert scale.

**Figure 12:**
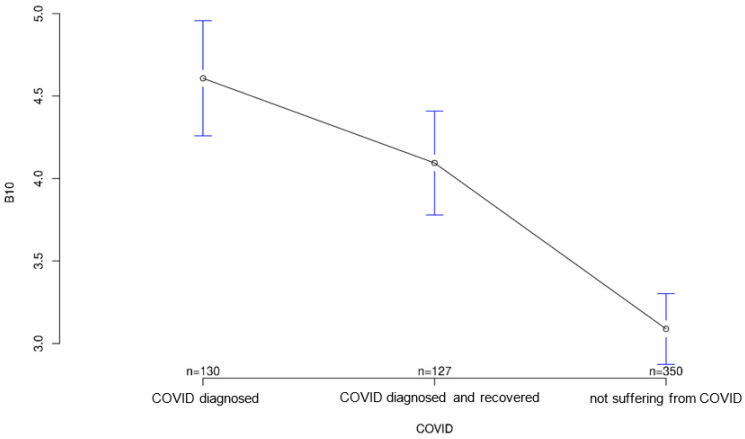
Average response to question 10 of the COVID-specific questionnaire: “Has your attitude toward outpatient medical technology changed since the COVID-19 pandemic?” The *y*-axis shows the mean result of participants choice on the Likert scale.

All three groups tended to agree with the statement that it was important for sensory equipment to be aesthetically pleasing or as unobtrusive as possible. Group differences regarding this statement were not statistically significant (*p* = 0.547; Figure 7). The healthy group was least likely to agree that medical records should be available to treating physicians, but again the group differences were not significant (*p* = 0.194, Figure 8). Interestingly, it was the recovered group that was most willing to share contact tracing data with government agencies while having the least concern about data security (*p* = 0.028 and *p* = 0.001, Figure 9 and Figure 10, respectively). The differences in these two statements reached statistical significance. The recovered group was also most likely to agree that patients should make private co-payments for such sensor technology, but these differences were not significant (*p* = 0.176, Figure 11). However, both the COVID-affected and recovered groups were significantly more likely than the healthy group to agree that their attitudes toward medical technology had changed since the COVID 19 pandemic (*p* < 0.001, Figure 12).

### 3.4. Confounding Parameters

The influence of various confounding parameters on participants’ responses between all three groups (COVID, recovered, and healthy) was examined. An influence of gender, marital status, having minor children, smoking status, place of residence, state, and education level on some of the responses was found, but this influence was small. These influences were examined in terms of interactions between Groups 1–3 (COVID, recovered, and healthy, respectively) and sociodemographic indicators when answering the questions. Results showed that, of 136 analyses in which the results for the questionnaire scales (i.e., curiosity, technology anxiety, etc.) were compared to sociodemographic factors, only 19 showed significant differences, whereas 117 show no statistical differences (Table A1 in Appendix A). There was a significant interaction between Groups 1–3 and gender for technology anxiety (*p* = 0.043) and for statement 2 of the COVID-specific questions (*p* = 0.028). Furthermore, significant interactions between Groups 1–3 and marital status were evident for curiosity (*p* = 0.023), skepticism (*p* = 0.039), and statement 10 of the COVID-specific questions (*p* = 0.041). A significant interaction between Groups 1–3 and minor children was shown for interest (*p* = 0.011) and for statement 10 of the COVID-specific questions (*p* = 0.030). Smoking status also had an influence on some responses in Groups 1–3. There were significant interactions between Groups 1–3 and smoking status for accessibility (*p* = 0.008) and for statements 6 (*p* = 0.033) and 9 (*p* = 0.007) of the COVID-specific questions. For education level, a significant interaction with Groups 1–3 was only found for statement 7 of the COVID-specific questions (*p* = 0.028). In contrast, for place of residence, significant interactions between Groups 1–3 were demonstrated for curiosity (*p* = 0.021), interest (*p* = 0.021), and for statements 1 (*p* = 0.005), 2 (*p* = 0.035), and 10 (*p* = 0.010) of the COVID-specific questions. For place of living, significant interactions within Groups 1–3 were found for interest (*p* = 0.028) and accessibility (*p* = 0.012). Within Groups 1–3 there was no significant interaction found when comparing the age groups with the different scales. All *p*-values for the interactions are listed in Table A1 in the Appendix A. Although it is important to mention that the sociodemographic variety between Groups 1–3 had some influence on the responses to the surveyed questions, in the majority of cases, the demographic characteristics did not have a significant impact on the participants’ answers. This should be taken into account when interpreting the group differences.

The presence of various comorbidities, such as hypertension, respiratory disease, diabetes mellitus, and cardiovascular disease, had a significant influence on some, but not all, of the responses and generally increased the response tendencies of COVID-affected and recovered individuals.

## 4. Discussion

The study presented here investigated the hypothesis that being or having been ill increases acceptance of medical technology. For this purpose, current COVID-19 patients (Group 1), people who had recovered from COVID-19 (Group 2), and people with no previous or current COVID-19 disease (Group 3) completed a questionnaire on different aspects of medical technology acceptance. The responses confirmed the hypothesis in several aspects, e.g., people diagnosed with COVID-19 and those who had recovered from it were more curious about modern medical technology and had a higher interest in using it, whereas healthy people were at the same time more fearful and skeptical about technology and its monitoring potential. Understanding the acceptance factors for medical technology is important to improve the use and benefits of new technologies. A study with scoliosis patients showed that aesthetic factors in the design of corsets were an important contributor to acceptance [14]. A comparable result was seen in the study presented here, where all three groups placed importance on the used sensor technology either looking aesthetically pleasing or being as unobtrusive as possible. Product design is relevant to how people view a technology and their willingness to use it.

However, most studies published to date have investigated the acceptability of a particular medical technology only in patients who use the particular technology as part of their treatment, and not in healthy people. The present study fills this gap by also looking at healthy individuals. By comparing the opinions of healthy people with those of people who suffer from or have survived COVID-19, the work enables a conclusion to be drawn about preventive medicine. Personal experience of health and illness leads people to trust or distrust technologies and value intrusions into their privacy.

Previous research shows that the stigma is particularly high for obvious medical aids, such as wheelchairs, and is associated with depression and limitations of use in patients with recent spinal cord injuries [15]. The observations of the present study show that even healthy individuals can be skeptical of monitoring and new technologies. As the wheelchair example shows, acceptance toward medical technology is important because it can significantly improve people’s lives and overcome stigma by promoting autonomy and self-determination.

Another interesting aspect of this study is that individuals who had recovered from COVID-19 were more accepting of using data from wearables or smartphones to track contacts and had fewer data security concerns than current COVID sufferers or healthy individuals. This may reflect the fact that COVID-19 is a highly contagious infectious disease with a relatively high case fatality rate for at-risk populations, and that those who have recovered may better appreciate the value of contact tracing based on their own experience. A further line of inquiry would be to investigate whether the risk of infection and/or the severity of the disease play a role. Why actively ill people are less “open-minded” is not clear. It is conceivable that being ill itself is a greater psychological burden at this time and thus altruistic motives, which may be present in recovered individuals, are relegated to the background.

### Limitations

This study had several limitations. First, recruitment was conducted via social media, which alters the age composition of potential study participants compared with the general population. Data collection via social media also raises questions about misinformation in and through social media. Participants’ sources of information were not queried. In addition, the study excluded individuals who are technophobic and do not use computers or social media from participation. Finally, the recruitment method excluded ill persons if they were too ill to use a computer at the time of recruitment. However, because this study was designed to identify differences in technology acceptance between patients, former patients, and healthy individuals, these limitations are of rather minor importance. Furthermore, due to the diverse nature of participants there were many sociodemographic differences between Groups 1–3, making results of comparative analysis between these groups difficult to interpret. To determine the extent to which these differences impacted our comparative analysis, we cross-examined all groups and their respective sociodemographic categories. These results, presented in Section 3.4 and listed in Table A1 in the Appendix A, show that, of 136 analyses, only 19 showed significant differences. Although it is important to mention these sociodemographic differences as possible limitations, these results further strengthen the validity of the data and conclusions.

## 5. Conclusions

This study confirmed the initial hypothesis that being ill or having recently been seriously ill increases open-mindedness toward new technologies and acceptance of medical technology related to the respective illness, and that this acceptance slightly decreases after recovery. This should have implications for preventive approaches in medicine.

## Figures and Tables

**Table 1:** Demographic parameters of the study participants.

Parameter	Total (*n* = 607)	Group 1 COVID (*n* = 130)	Group 2 Recovered (*n* = 127)	Group 3 Healthy (*n* = 350)	*p*-Value
Gender *n* (%)					<0.001
Female	448 (73.8)	59 (45.4)	67 (52.8)	322 (92.0)	
Male	155 (25.5)	70 (53.7)	59 (46.5)	26 (7.4)	
Divers	4 (0.7)	1 (0.8)	1 (0.8)	2 (0.6)	
Age group					<0.001
18–24 years	166 (27.4)	15 (11.5)	18 (14.2)	133 (38.0)	
25–34 years	251 (41.4)	33 (24.4)	46 (36.2)	172 (49.1)	
35–44 years	73 (12.0)	25 (19.2)	21 (16.5)	27 (7.7)	
45–54 years	56 (9.2)	20 (15.4)	21 (16.5)	15 (4.3)	
55–64 years	26 (4.3)	10 (7.7)	13 (10.2)	3 (0.9)	
65–74 years	27 (4.4)	19 (14.6)	8 (6.3)	0 (0.0)	
75–84 years	7 (1.2)	7 (5.4)	0 (0.0)	0 (0.0)	
85 years or older	1 (0.2)	1 (0.8)	0 (0.0)	0 (0.0)	
Marital status *n* (%)					<0.001
Single	218 (35.9)	34 (26.2)	34 (26.8)	150 (42.9)	
married	258 (42.5)	49 (37.7)	56 (44.1)	153 (43.7)	
cohabiting	74 (12.2)	22 (16.9)	23 (18.1)	29 (8.3)	
divorced/separated	44 (7.2)	18 (13.9)	12 (9.4)	14 (4.0)	
widowed	9 (1.5)	7 (5.4)	2 (1.6)	0 (0.0)	
other	4 (0.7)	0 (0.0)	0 (0.0)	4 (1.1)	
Has minor children *n* (%)	174 (28.7)	34 (25.0)	32 (24.6)	108 (29.8)	0.380
Smoking status Yes *n* (%)	131 (21.6)	46 (33.8)	35 (26.9)	50 (13.8)	<0.001
Educational level *n* (%)					<0.001
University degree	205 (33.8)	56 (43.1)	51 (40.2)	98 (28.0)	
Fachabitur (vocational baccalaureate)/Abitur (university entrance qualification)	221 (36.4)	32 (24.6)	32 (25.2)	157 (44.9)	
Realschulabschluss (general certificate of secondary education)	130 (21.4)	29 (22.3)	33 (26.0)	68 (19.4)	
Hauptschule (secondary school)/Volksschule (adult education college)	30 (4.9)	10 (7.7)	8 (6.3)	12 (3.4)	
no graduation	9 (1.5)	3 (2.3)	3 (2.4)	3 (0.9)	
other	12 (2.0)	0 (0.0)	0 (0.0)	12 (3.4)	
Place of residence *n* (%)					<0.001
Big city	305 (50.2)	55 (42.3)	58 (45.7)	192 (54.9)	
Medium-sized town	170 (28.0)	58 (44.6)	29 (22.8)	83 (23.7)	
Small town	100 (16.5)	10 (7.7)	35 (27.6)	55 (15.7)	
Rural community	32 (5.3)	7 (5.5)	5 (3.9)	20 (5.7)	
Place of living *n* (%)					<0.001
Baden-Württemberg					
Bavaria	53 (8.7)	11 (8.5)	7 (5.5)	35 (10.0)	
Berlin	48 (7.9)	15 (11.5)	7 (5.5)	26 (7.4)	
Brandenburg	26 (4.3)	3 (2.3)	8 (6.3)	15 (4.3)	
Bremen	6 (1.0)	3 (2.3)	2 (1.6)	1 (0.3)	
Hamburg	3 (0.5)	0 (0.0)	2 (1.6)	1 (0.3)	
Hesse	86 (14.2)	14 (10.8)	11 (8.7)	61 (17.4)	
Mecklenburg-West. P.	58 (9.6)	7 (5.4)	6 (4.7)	45 (12.9)	
Lower Saxony	16 (2.6)	4 (3.1)	9 (7.1)	3 (0.9)	
North Rhine-Westphalia	80 (13.2)	34 (26.2)	15 (11.8)	31 (8.9)	
Rhineland-Palatinate	145 (23.9)	23 (17.7)	43 (33.9)	79 (22.6)	
Saarland	11 (1.8)	2 (1.5)	2 (1.6)	7 (2.0)	
Saxony	1 (0.2)	0 (0.0)	0 (0.0)	1 (0.3)	
Saxony-Anhalt	3 (0.5)	1 (0.8)	1 (0.8)	1 (0.3)	
Schleswig-Holstein	41 (6.8)	11 (8.5)	10 (7.9)	20 (5.7)	
Thuringia	4 (0.7)	1 (0.8)	1 (0.8)	2 (0.6)	
Austria	4 (0.7)	1 (0.8)	1 (0.8)	2 (0.6)	
Switzerland	5 (0.8)	0 (0.0)	0 (0.0)	5 (1.4)	
other place of living	17 (2.8)	0 (0.0)	2 (1.6)	15 (4.3)	

## Data Availability

The data used for all statistical analysis and calculations are stored in the research data repository PsychArchives and can be found here: https://hdl.handle.net/20.500.12034/4223 (accessed on 25 July 2021). Data storage was performed as stated in the ethical application under the number MSH-2021/121.

## Appendix A

**Table A1:** *p*-values of interactions between Group 1 (COVID), Group 2 (Recovered), and Group 3 (Healthy) and sociodemographic indicators for all dependent variables. ANOVA comparing Groups 1–3 to the different scales of the TUI questionnaire.

	Interactions							
	Group 1–3 Group Gender	Group 1–3 Group Age Group	Group 1–3 Group Marital Status	Group 1–3 Group Has Minor Children	Group 1–3 Group Smoking Status	Group 1–3 Educational Level	Group 1–3 Group Place of Residence	Group 1–3 Group Place of Living
Curiosity	0.545	0.965	**0.023**	0.214	0.216	0.171	**0.021**	0.320
Fear of technology	**0.043**	0.330	0.121	0.975	0.113	0.774	0.453	0.860
Interest	0.418	0.274	0.242	**0.011**	0.210	0.297	**0.021**	**0.028**
Usefulness	0.443	0.828	0.100	0.365	0.422	0.193	0.175	0.326
Skepticism	0.601	0.286	**0.039**	0.928	0.608	0.858	0.510	0.973
Usability	0.083	0.765	0.353	0.371	0.451	0.531	0.161	0.913
Accessibility	0.139	0.824	0.696	0.175	**0.008**	0.364	0.338	**0.012**
COVID-specific statement 1	0.319	0.872	0.998	0.321	0.476	0.112	**0.005**	0.302
COVID-specific statement 2	**0.028**	0.856	0.147	0.957	0.125	0.360	**0.035**	0.364
COVID-specific statement 3	0.451	0.745	0.540	0.583	0.603	0.514	0.351	0.439
COVID-specific statement 4	0.657	0.993	0.169	0.622	0.335	0.519	0.110	0.067
COVID-specific statement 5	0.512	0.669	0.523	0.519	0.693	0.674	0.149	0.093
COVID-specific statement 6	0.422	0.924	0.115	0.784	**0.033**	0.273	0.503	0.370
COVID-specific statement 7	0.127	0.614	0.325	0.586	0.491	**0.028**	0.153	0.513
COVID-specific statement 8	0.728	0.397	0.077	0.164	0.717	0.376	0.328	0.800
COVID-specific statement 9	0.396	0.076	0.851	0.631	**0.007**	0.205	0.762	0.512
COVID-specific statement 10	**0.004**	0.835	**0.041**	**0.030**	0.579	0.622	**0.010**	0.170

Significant *p*-values are marked in bold.
